# In silico analysis of a novel hypothetical protein (YP_498675.1) from *Staphylococcus aureus* unravels the protein of tryptophan synthase beta superfamily (Try-synth-beta_ II)

**DOI:** 10.1186/s43141-023-00613-7

**Published:** 2023-11-23

**Authors:** Vivian Chakma, Dhirendra Nath Barman, Shuvo Chandra Das, Anwar Hossain, Monira Binte Momin, Maisha Tasneem, Shipan Das Gupta

**Affiliations:** Department of Biotechnology and Genetic Engineering, Noakhali Science and Technology University, Noakhali, 3814 Bangladesh

**Keywords:** *Staphylococcus aureus*, Hypothetical protein, Homology modeling, Staphyloferrin B, SbnA domain

## Abstract

**Background:**

*Staphylococcus aureus* is a gram-positive spherical bacteria and the most common cause of nosocomial infections in the world. Given its clinical significance, the genome sequence of *S. aureus* has been elucidated to enhance our comprehension of its lifestyle and pathogenicity. The research aimed to summarize a potential hypothetical protein that may play an important role in *S. aureus* virulence and pathogenicity, covering its anticipated structure, probable biological functions, and importance in this context.

**Results:**

A hypothetical protein, YP_498675.1 with 281 amino acid residues of *S. aureus*, was chosen for analysis and modeling by several bioinformatics tools and databases in this work. According to primary and secondary structure analyses, YP_498675.1 is a stable hydrophilic protein with a significant proportion of *α*–helices. Subcellular localization predictions by CELLO, PSORTb, and SOSUI server indicate that it is a cytoplasmic protein. NCBI-CDD, Pfam, and InterProScan functional genomics research revealed that the hypothetical protein may include the pyridoxal phosphate (PLP)-dependent 2, 3-diaminopropionate biosynthesis protein SbnA domain. In the homology modeling method, the HHpred server was employed to create its 3D structure using the template structure of a Staphyloferrin B precursor biosynthetic enzyme SbnA bound to PLP (PDB ID: 5D84_A), an X-ray diffraction model having 100% sequence identity with the hypothetical protein. After energy minimization, several quality assessments and validation factors determined that the generated protein model was reliable and of reasonable quality.

**Conclusion:**

The present study has characterized and functionally annotated the hypothetical protein YP_498675.1 of *S. aureus*. Further experimental validation would aid in determining the actual function of YP_498675.1 as well as confirm the protein’s value as a therapeutic target.

## Background 

Scientists may gather massive volumes of data in a relatively short period of time using next-generation sequencing (NGS). As more organisms are being sequenced, the challenge of assigning functions to genes is increasing [1, 2]. In many organisms, the molecular functions of more than 30% of proteins are unknown termed “Hypothetical Proteins (HP)” [3]. In silico characterization of HP aids in the determination of three-dimensional (3D) structures, which can reveal new domains and motifs, pathways, protein networks, and other information [4–6]. Furthermore, novel HP may also serve as potential biomarkers and pharmacological targets for drug design, discovery, and screening [7, 8]. The functions of hypothetical proteins from various pathogenic bacteria have been successfully annotated using a variety of bioinformatics strategies [9–15]. Sequence similarity, phylogenetic analysis, protein–protein interactions, protein–ligand interactions, active site residue similarity, conserved domains, motifs, phosphorylation sites, and gene expression patterns were all used to achieve this [16]. Although *S. aureus* have been there since the beginning of time, they were first discovered as a disease causing agent in the nineteenth century. In 1880, Alexander Ogston first observed grape-like clusters of bacteria in pus from a surgical abscess in a knee joint and named them *Staphylococcus* [17]. In 1884, German doctor Friedrich Julius Rosenbach was able to cultivate the organisms in pure culture and classify them according to how their color creation [17]. S*taphylococcus aureus* is a gram-positive, spherical bacterium with a diameter of around 1 μm, which are responsible for a wide range of clinical illnesses [18]. It is often found as a commensal associated with skin, skin glands, and mucous membranes, particularly in the nose of healthy individuals [19]. It has been estimated that approximately 20–30% of the general population are *S. aureus* carriers [20]. The most common way of transmission is through contaminated hands**.***S. aureus* is one of the main causes of hospital and community-acquired infections, which can result in serious consequences [21]. Circulation system, skin, delicate tissues, and lower respiratory tracts are all affected by nosocomial *S. aureus* diseases. *S. aureus* can also lead to bone, joint, and endovascular diseases [22]. Infections with *S. aureus* can result in ventilator assisted pneumonia as well as central venous catheter-associated bacteremia. Moreover, it causes serious deep-seated infections, such as endocarditis and osteomyelitis [23]. Along with the infections mentioned above, *S. aureus* frequently causes toxin-mediated diseases such as toxic shock syndrome, scalded skin syndrome, and staphylococcal foodborne illnesses (SFD) [18]. Dairy cow’s milk in Bangladesh has been identified to contain Methicillin-resistant *S. aureus* (MRSA) that may lead to septicemia, pneumonia, and dermatitis [24]. The emergence of antibiotic resistance in *S. aureus* demands new strategies for treating infections caused by this pathogen. One potential avenue is the development of a vaccine or drug targeting a HP unique to *S. aureus*, which could help to overcome the limitation posed by antibiotic resistance. By thoroughly characterizing the properties and function of a HP, researchers can gain crucial knowledge about its potential as a vaccine candidate and explore its effectiveness in eliciting an immune response capable of combating *S. aureus* infection.

The genome of *S. aureus* measures about 2.82 Mp in size, with a mean GC content of 32.90%. It has so far been discovered with 2872 genes, and 2767 proteins. To date, a total number of 1511 proteins of *S. aureus* have been identified with no known function. About half of the genomic proteins in reference strain *S. aureus* NCTC 8325 are hypothetical [25]. There is a high demand to characterize the hypothetical proteins because annotating these proteins may result in new treatment targets [25]. The hypothetical protein (YP_498675.1) from *S. aureus* was used in this work since its structural characteristics are unknown, but its core amino acid sequence is known. The goal of this study was to investigate the physiochemical and secondary structural characteristics of the putative *S. aureus* protein (YP_498675.1), construct its first three-dimensional (3D) model through homology modeling, and conduct functional and comparative genomics research using Basic Local Alignment Search Tool for proteins (BLASTp) and multiple sequence alignment (MSA) analysis. The current study aims to enhance our understanding of the functional roles performed by members of the staphylococci community, thereby offering valuable insights into potential therapeutic targets.

## Materials and methods

### Procedures for the filtration and selection of a specific hypothetical protein

**Fig. 1:**
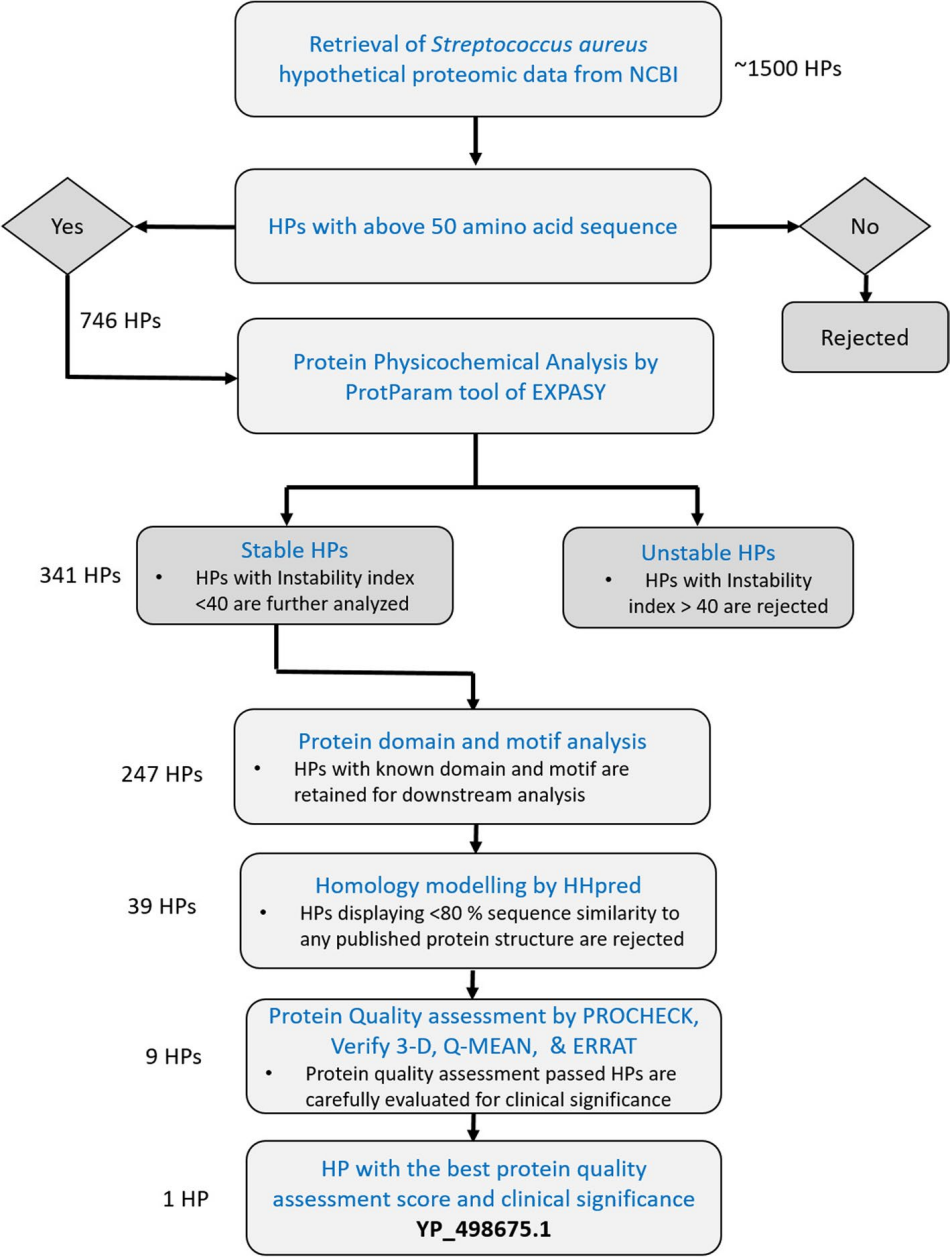
Workflow of the filtration and selection process of the hypothetical protein (YP_498675.1). Number of HP at each step of the filtration process are indicated on the left side

The proteomic data of *S. aureus* was sourced from National Center for Biotechnology Information (NCBI) (http://www.ncbi.nlm.nih.gov/) database. Initially, approximately 1500 HP from *S. aureus* were chosen for subsequent in silico analysis. Exclusion criteria were applied to eliminate HP with amino acid sequences shorter than 50 residues, as proteins below this length are known to exhibit compromised folding characteristics. Out of the initial pool of 746 HP with amino acid sequences longer than 50 residues, their protein physicochemical properties were analyzed using ProtParam tool. Approximately 405 HP were excluded from further analysis due to exhibiting unstable characteristics according to ProtParam results. The remaining stable HP (341) underwent subcellular localization analysis using CELLO v2.5, PSoRTb, SOSUI, and PSLpred. HP that consistently displayed the same result across all the four tools were then selected for protein domain and motif prediction. After undergoing subcellular localization analysis and subsequent protein domain and motif screening steps, a total of 94 HP were rejected from further analysis. In the subsequent step, we employed homology modeling to predict the three-dimensional (3D) structure of HP. HP displaying less than 80% sequence similarity to any published protein structure are rejected for downstream analysis. At this stage of screening, we were able to delimit the HP number to 39. These predicted models were then subjected to protein quality assessment to evaluate their reliability and accuracy. As a result of this screening process, the number of HP was successfully reduced to below ten, as several models did not meet the quality assessment criteria and were therefore excluded from further consideration. From this narrowed-down selection, we carefully considered the clinical significance and function of all predicted HP, and the best-performing HP, YP_498675.1, consisting of 281 amino acids, was chosen as the representative for our manuscript. The overall workflow of this screening process is depicted in Fig. 1.

### Physicochemical properties analysis

The ExPASy ProtParam [26] (https://web.expasy.org/protparam/) tool was used to characterize HP in terms of their physicochemical features. Parameters such as molecular weight, aliphatic index (AI), extinction coefficients, amino acid composition, grand average of hydropathy (GRAVY), isoelectric point (pI), and estimated half-life were analyzed.

### Prediction of protein subcellular localization

The putative subcellular localization of the HP (YP_498675.1) was determined by CELLO v.2.5 (http://cello.life.nctu.edu.tw/) [27], an analysis based on a two-level support vector prediction system (SVM). Subcellular localization predicted by CELLO was further correlated with the result of PSoRTb (https://www.psort.org/psortb/) [28], SOSUI (https://harrier.nagahama-i-bio.ac.jp/sosui/mobile/) [29], and PSLpred (https://webs.iiitd.edu.in/raghava/pslpred/submit.html) [30]. SOSUI discriminates between soluble and transmembrane proteins by calculating the average hydrophobicity of protein. In contrast, PSORTb and PSLpred predict subcellular localization of prokaryotic proteins on the basis of various features, e.g., amino acid and dipeptide composition, composition of physicochemical properties, and evolutionary information of PSI-BLAST.

### Identification of protein domain and motif

NCBI Conserved Domain Search (NCBI CD-Search) (https://structure.ncbi.nlm.nih.gov/Structure/cdd/wrpsb.cgi) [31], Protein families database (Pfam 34.0) (http://pfam.xfam.org/) [32], and InterProScan5 (http://www.ebi.ac.uk/Tools/services/web/toolform.ebi?tool=iprscan5&sequence=uniprot:KPYM_HUMAN) [33] were used for domain analysis of YP_498675.1. We utilized the Conserved Domain Database (CDD) through Reverse Position-Specific BLAST (RPS-BLAST) and the InterProscan tool for our analyses. Pfam, a protein family database, employed hidden Markov models (HMMs) to generate annotations and multiple sequence alignments. To identify the protein sequence motif, we employed the MOTIF search tool (https://www.genome.jp/tools/motif/) InterProscan tool. Pfam is a protein family database that uses hidden Markov models (HMMs) in order to generate annotations and multiple sequence alignments. To determine the protein sequence motif, MOTIF Search (https://www.genome.jp/tools/motif/) tool was used [34].

### Protein family and phylogenetic tree analysis

In order to identify the homologs of the HP (YP_498675.1), a protein-BLAST (BLASTp) (https://blast.ncbi.nlm.nih.gov/Blast.cgi?PAGE=Proteins) [35] from NCBI (National Center for Biotechnology Information) against the non-redundant database with default parameters was performed. This approach is based on the local alignment of protein sequence to find similar proteins. CLC Sequence Viewer version 8 (https://clc-sequence-viewer.software.informer.com/8.0/) was employed to perform multiple sequence alignment and generate a phylogenetic tree for a specific subset of sequences.

### Secondary structure prediction

Two-dimensional structure of the YP_498675.1 protein was determined using SOPMA (self-optimized prediction method with alignment) (https://npsa-prabi.ibcp.fr/cgi-bin/npsa_automat.pl?page=/NPSA/npsa_sopma.html) [36] and PSI-PRED (Position Specific Iterated – BLAST) (http://bioinf.cs.ucl.ac.uk/psipred/) [37]. Result from SOPMA analysis was correlated with the result of PSI-PRED.

### Homology modeling

HHpred server (https://toolkit.tuebingen.mpg.de/tools/hhpred) [38] was used to determine the 3D structure of YP_498675.1 and the performance of this determination was based on the pairwise comparison profile of hidden Markov models (HMMs). HHpred server allows to search a wide choice of databases, such as the PDB, SCOP, Pfam, SMART, COGs, and CDD. The quality of each detected template has been projected based on aspects of the target-template alignment. The template protein of a Staphyloferrin B precursor biosynthetic enzyme SbnA bound to PLP (PDB ID: 5D84_A) with 100% sequence identity to our hypothetical protein was chosen for homology modeling. UCSF Chimera 1.16 was employed to visualize the 3D model structure [39].

### Quality assessment

Structural evaluations of the protein model were performed by using several programs called PROCHECK (https://www.ebi.ac.uk/thornton-srv/software/PROCHECK/) [40], Verify 3D (https://servicesn.mbi.ucla.edu/Verify3D/) [41], ERRAT [42], and Qualitative Model Energy Analysis (QMEAN) (https://swissmodel.expasy.org/qmean/) [43] programs of ExPASy server of SWISS-MODEL Workspace. PROCHECK performs various assessments including the generation of a Ramachandran plot and measurement of torsion angles, surface areas, bond angle, and atomic distances [40]. The accuracy of the overall fold/structure, as well as inaccuracies in localized regions and stereo chemical characteristics such as bond lengths and angles, were all checked model evaluation. Verify 3D determines the compatibility of an atomic model (3D) with its own amino acid sequence (1D) by assigning a structural class based on its location and environment (alpha, beta, loop, polar, nonpolar, etc.) and comparing the results to good structures [44]. A score above 80% on Verify 3D indicates good quality for protein structures. QMEAN, short for Qualitative Model Energy Analysis, is a composite scoring function describing the major geometrical aspects of protein structures [45]. ERRAT stands for “Evaluation of Protein Structure by Ramachandran Plot Assessment.” The ERRAT score is a metric for assessing the accuracy and quality of protein models. By evaluating the statistical significance of the difference between predicted and expected atomic interactions, the ERRAT score evaluates the model’s compatibility with known protein structures [42]. These analyses provide valuable insights into the quality and accuracy of the protein models, ensuring their reliability for further analysis and interpretation.

### Energy minimization of the model structure

The 3D structure of the hypothetical protein YP_498675.1 was refined by performing YASARA energy minimization server [46]. In order to perform the protein energy minimization of the PDB file of the three-dimensional protein, model structure was uploaded. The server minimizes the energy required by providing a more precise and stable 3D structure of the desired protein (YP_498675.1).

### Active site analysis

Computed atlas of surface topography of proteins (CASTp) (http://sts.bioe.uic.edu/castp/) server was used to find out the ligand binding sites of the hypothetical protein YP_498675.1. CASTp obtains the topographical features of a protein in a detailed, comprehensive, and quantitative manner. CASTp predicts active pockets located on protein surfaces and in the interior site of the 3D structure, the regions and key residues of protein which interact with ligands. As a result, it has become an essential tool for predicting regions and key residues of protein which interact with ligands [47]. The CASTp result was also displayed using PyMOL software [48].

## Results

### Analysis of physicochemical properties and sub-cellular localization

The theoretical physiochemical features of the hypothetical protein YP_498675.1 were analyzed using ExPASy’s ProtParam server (Table 1). The protein was predicted to be consisting of 281 amino acids, with a molecular weight of 30,872.44 Daltons and an isoelectric point (PI) of 5.78. It is well established that proteins with an instability index below 40 are considered stable, whereas those with a value exceeding 40 are deemed unstable [49]. In the case of the analyzed hypothetical protein YP_498675.1, its instability index was calculated to be 29.48, indicating that it falls within the stable range. The negative grand average of hydropathicity (GRAVY) index of − 0.119 is indicative of a hydrophilic and soluble protein. The most abundant amino acid residue was found to be isoleucine (35), followed by glycine (24) and alanine (21). The lowest was found as cysteine (2). The sequence had 35 negatively charged residues (aspartic acid + glutamic acid) and 29 positively charged residues (arginine + lysine). The atomic composition comprises of 4381 atoms having molecular formula of protein C_1375_H_2211_N_369_O_417_S_9_.

**Table 1:** Analysis of physiochemical properties of the YP_498675.1 using ProtParam

Descriptions	Value
Number of amino acids	281
Molecular weight	30,872.44 KDa
Theoretical pI	5.78
Total number of negatively charged residues	35
Total number of positively charged residues	29
Ext. coefficient	33,015 M^−1^ cm^−1^
Instability index	29.48
Aliphatic index	102.38
Grand average of hydropathicity (GRAVY)	− 0.119

The function of a protein is greatly influenced by its location within the cell. Predicting the subcellular localization of unknown proteins would be beneficial because different cellular locations represent different functions. This information could aid in the study of disease mechanisms and the development of new drugs [27, 28]. Our query protein’s (YP_498675.1) subcellular localization was predicted to be a cytoplasmic. Subcellular location of YP_498675.1 was analyzed by CELLO and authenticated by PSORTb v3.2.0, SOSUIGramN, and PSLpred server.

#### Secondary structure prediction of YP_498675.1

**Fig. 2:**
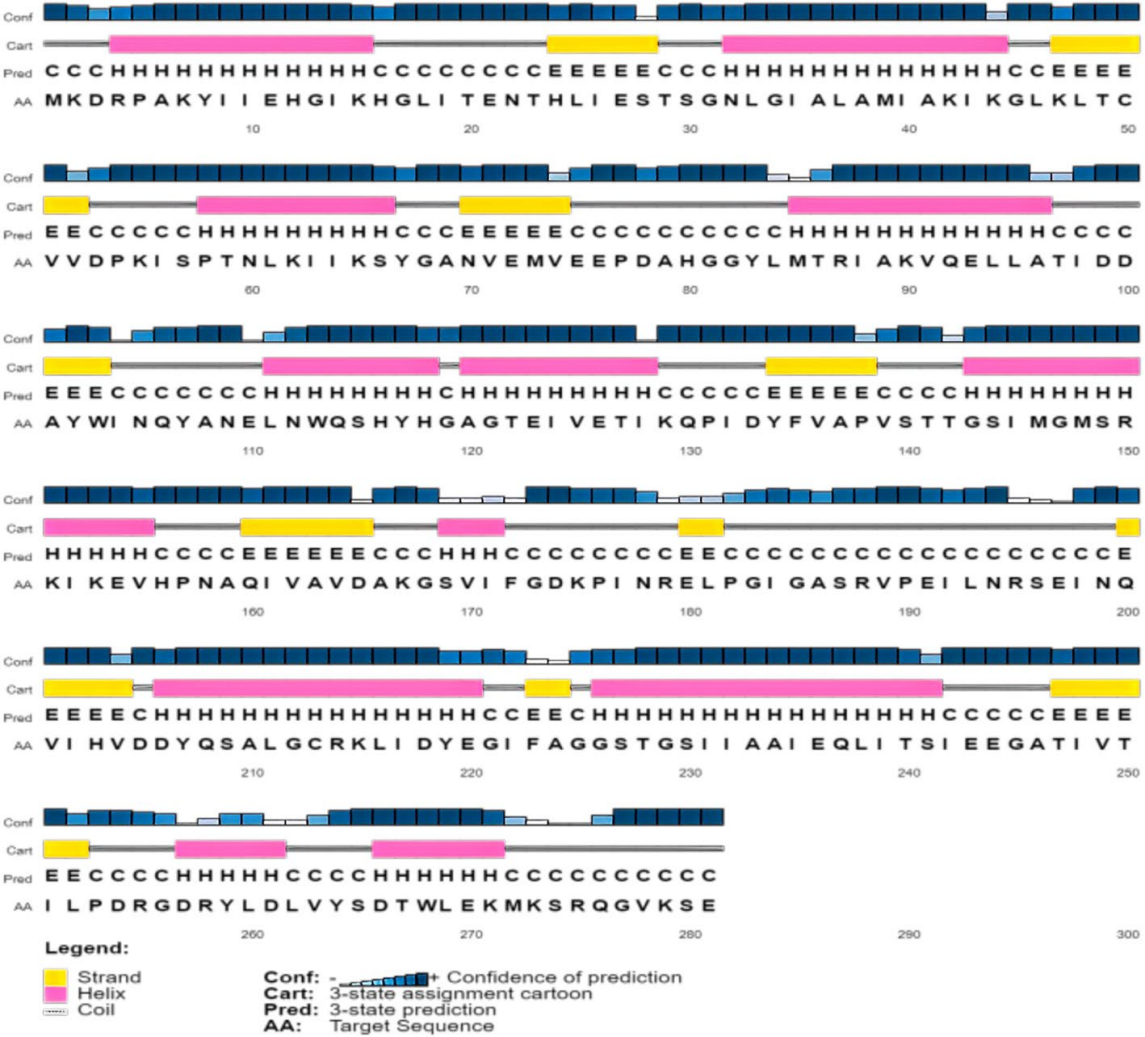
Protein secondary structure prediction of the (YP_498675.1) using the PSI-PRED server. This graphical representation has four different sections. The first section is made up of bars of varying heights. The height of the bar is proportional to the confidence score. The pink color represents the alpha helix, the yellow color represents beta sheets or strands, and the gray color represents coils in the second section. The coil connects a particular alpha helix with the particular beta sheets. The third section contains an alphabetic representation, which denotes the secondary structure of a protein; Here, E, H, and C are used for beta sheets, alpha helixes, and coils, respectively. The arrangement of amino acids is presented alphabetically in the final section

PSI-PRED and SOPMA servers were used to investigate the secondary structure of the YP_498675.1. The SOPMA secondary prediction server analysis revealed the proportions of alpha helix, beta turn, extended strand, and the random coil of the protein as 40.57%, 13.17%, 18.15%, and 28.11%, respectively. Similar results were also observed in PSI-PRED tool (Fig. 2).

#### Prediction of protein family by domain and motif analysis

NCBI-CD Search, Pfam, and InterProScan annotation tools were used to identify conserved domains and potential function of the YP_498675.1. The specific hit explored by conserved domain (CD) search tool predicted the query protein belongs to tryptophan synthase beta superfamily (fold type II) (Try-synth-beta_ II). Protein of this family is pyridoxal phosphate (PLP)-dependent enzyme covers 1 to 269 amino acid residues with an *E*-value of 4.38e-168 of our protein sequence. The result of CD search analysis was found to be comparable with the result of two other domain searching tools namely InterProscan and Pfam. InterProscan covers 1 to 254 amino acid residues with an *E*-value of 1.4e-46. The Pfam tool predicted the tryptophan synthase beta superfamily covers 1 to 254 amino acid residues with an *E*-value of 1.5e-46. MOTIF server predicted pyridoxal phosphate (PLP)-dependent enzyme at the position of 1 to 254 amino acid residues with an *E*-value of 1.5e-46.

#### Comparative genomics analysis of YP_498675.1 using multiple sequence alignment and phylogeny

**Fig. 3:**
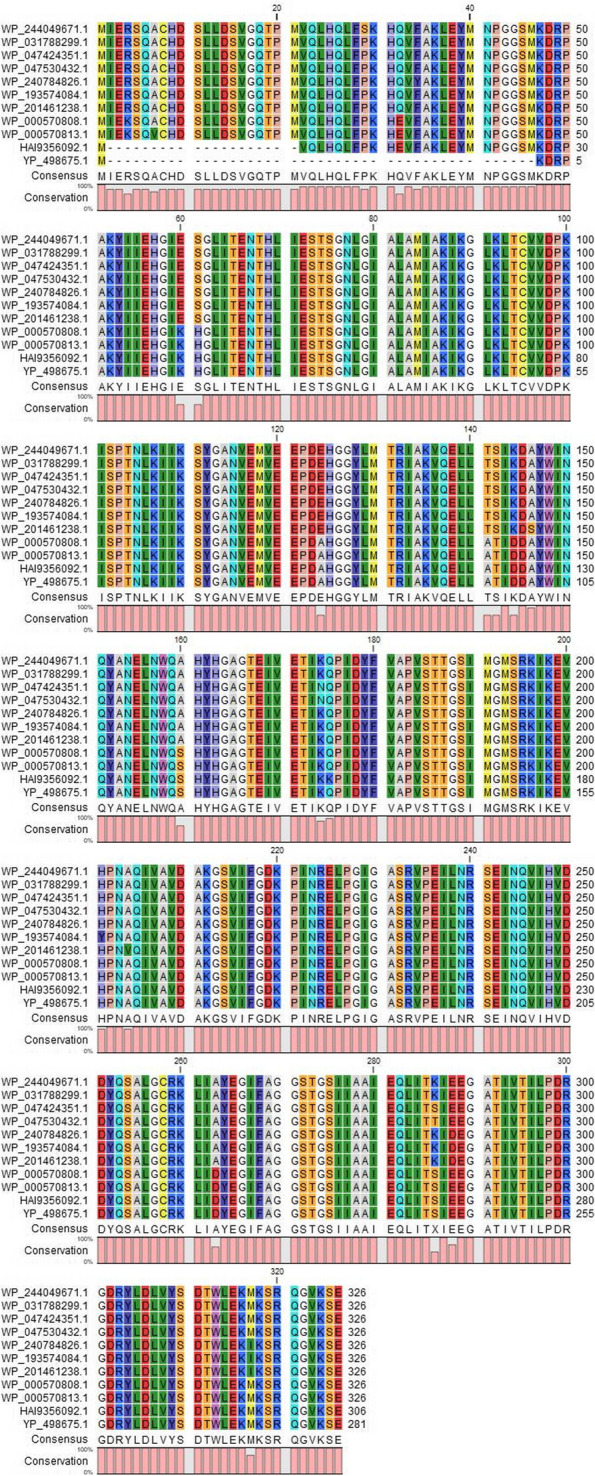
MSA analysis among the different types of 2, 3-diaminopropionate biosynthesis protein SbnA with the YP_498675.1. Sources for the sequences: row 1 and 2 *Staphylococcus argenteus*; row 3 and 4 *S. schweitzeri*; row 5 and 7 *S. roterodami*; row 6 *S. singaporensis*; row 8 and 9 *S. aureus*; row 10 *Escherichia coli*; last row target protein (YP_498675.1)*.* MSA indicates multiple sequence alignment (generated by CLC Sequence Viewer Version 8)

**Fig. 4:**
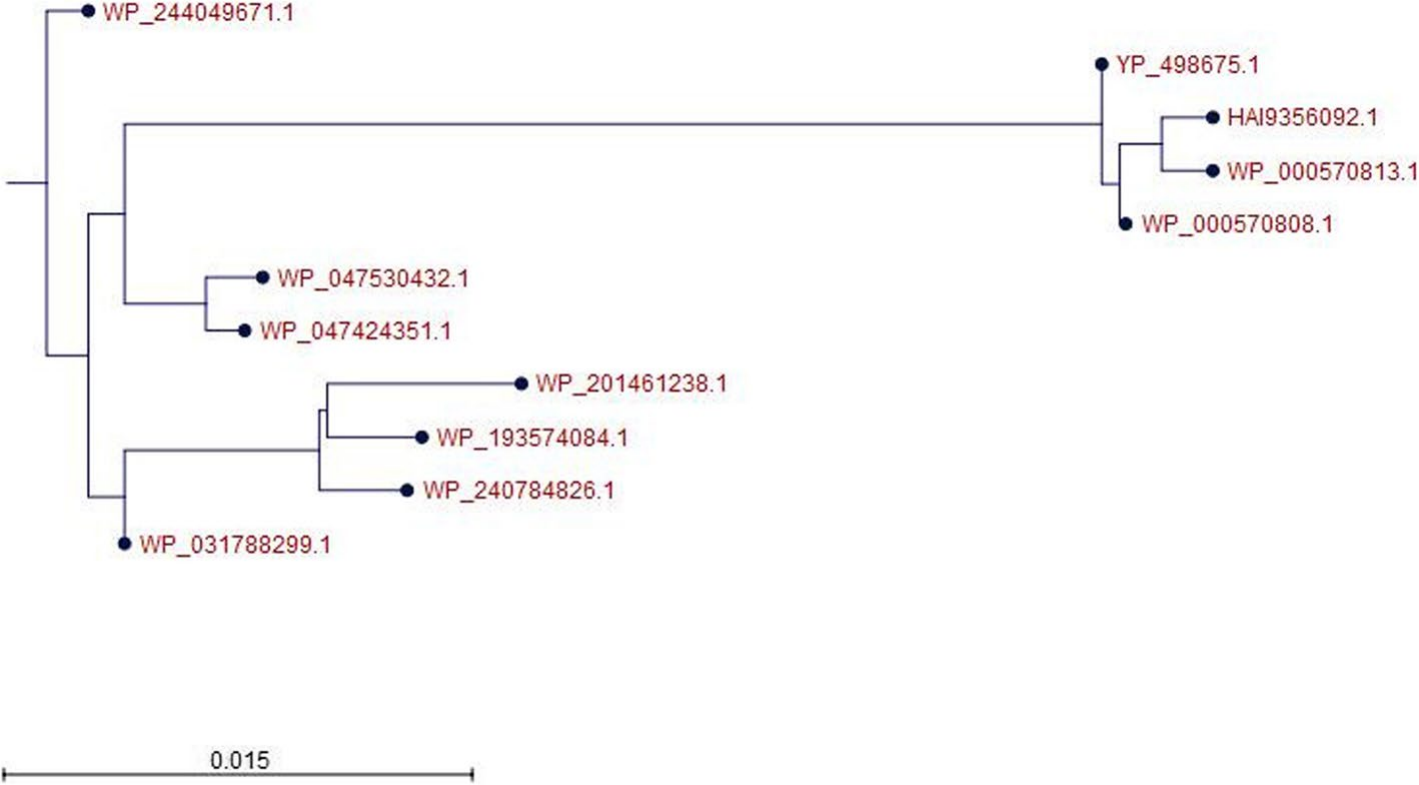
Phylogenetic tree illustrating evolutionary relationship of YP_498675.1 with closely related proteins. The tree was generated using CLC Sequence Viewer Version 8. Here, the scale bar estimates sequence divergence, and amount of genetic change is represented by the line segment with the number (0.015)

The BLASTp search against the non-redundant database showed homology (up to 100% sequence similarity) with other known type of tryptophan synthase beta superfamily protein from different *Staphylococcus* species (Table 2). A total of 10 selected protein sequences along with the target sequence were retrieved from BLASTp analysis for multiple sequence alignment (MSA). MSA was completed using the CLC sequence viewer in order to observe the conserved and dissimilar residues among the homologs (Fig. 3). Using the same data, a phylogenic tree was created (Fig. 4). The target protein along with the three other proteins from *Staphylococcus* species (WP_000570813.1 and WP_0000808.1) and *Escherichia coli* (HAI9356092.1) appear to have common ancestor with the WP_047424351.1 and WP_047530432.1 proteins of *S. schweitzeri*. The scale bar estimates sequence divergence, and amount of genetic change is represented by the line segment with the number (0.015).

**Table 2:** Identification of homologs of YP_498675.1 through protein BLASTp search analysis

Description	Scientific name	Total score	Query cover	E value	Per. ident	Accession
2,3-diaminopropionate biosynthesis protein SbnA (Staphylococcus)	*Staphylococcus*	575	100%	0	100	WP_000570808.1
2,3-diaminopropionate biosynthesis protein SbnA (Staphylococcus)	*Staphylococcus*	575	100%	0	100	WP_000570813.1
TPA: 2,3-diaminopropionate biosynthesis protein SbnA (*Escherichia coli*)	*Escherichia coli*	572	100%	0	99.64	HAI9356092.1
2,3-diaminopropionate biosynthesis protein SbnA (*Staphylococcus argenteus*)	*Staphylococcus argenteus*	557	100%	0	96.8	WP_244049671.1
2,3-diaminopropionate biosynthesis protein SbnA (*Staphylococcus schweitzeri*)	*Staphylococcus schweitzeri*	556	100%	0	96.8	WP_047424351.1
2,3-diaminopropionate biosynthesis protein SbnA (*Staphylococcus argenteus*)	*Staphylococcus argenteus*	556	100%	0	96.8	WP_031788299.1
2,3-diaminopropionate biosynthesis protein SbnA (*Staphylococcus schweitzeri*)	*Staphylococcus schweitzeri*	556	100%	0	96.44	WP_047530432.1
2,3-diaminopropionate biosynthesis protein SbnA (*Staphylococcus roterodami*)	*Staphylococcus roterodami*	553	100%	0	96.09	WP_240784826.1
2,3-diaminopropionate biosynthesis protein SbnA (*Staphylococcus singaporensis*)	*Staphylococcus singaporensis*	551	100%	0	95.73	WP_193574084.1
2,3-diaminopropionate biosynthesis protein SbnA (*Staphylococcus roterodami*)	*Staphylococcus roterodami*	551	100%	0	95.37	WP_201461238.1

### Three-dimensional structure determination and model quality assessment

**Fig. 5:**
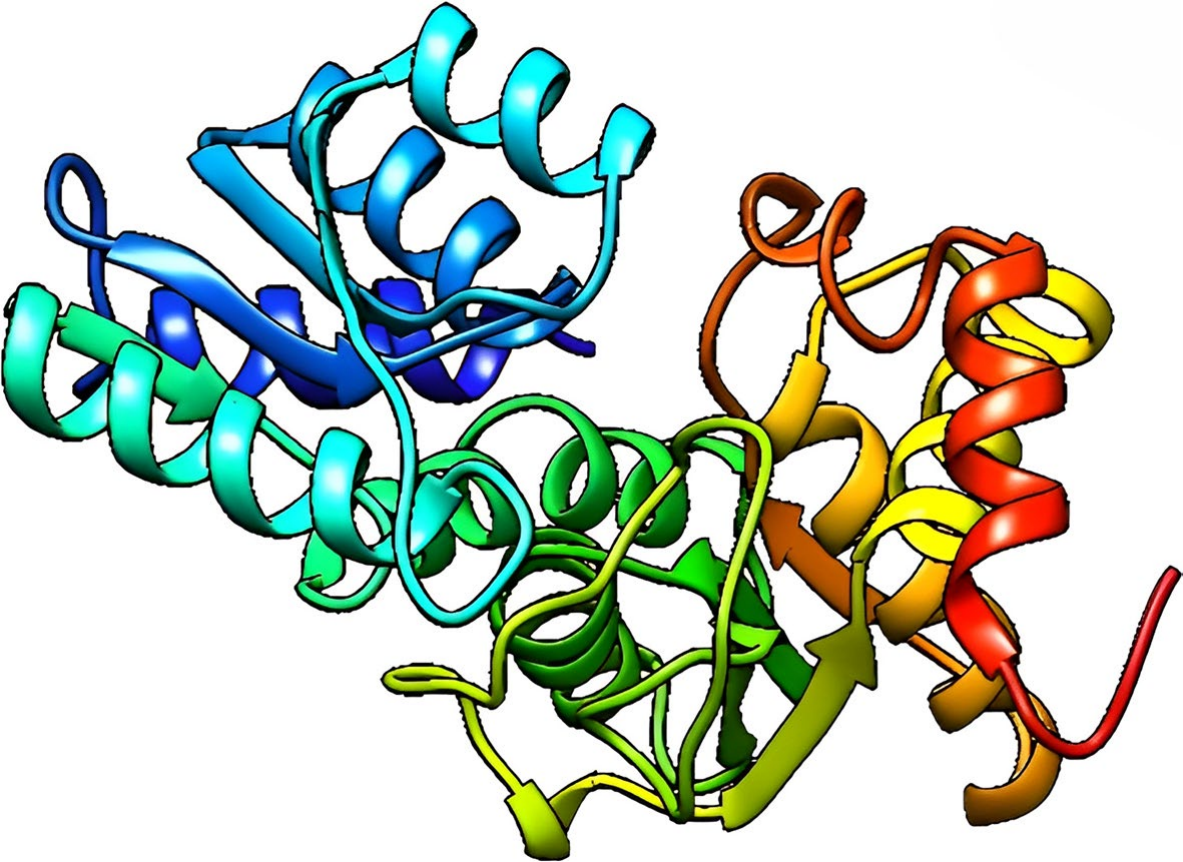
Predicted three-dimensional structure of the YP_498675.1 (visualized by UCSF Chimera 1.16)

The query sequence was submitted into the HHpred server for protein homology detection and structure prediction [38]. The 3D structure of YP_498675.1 was determined using the template structure of the Staphyloferrin B precursor biosynthetic enzyme SbnA bound to PLP (PDB ID: 5D84_A) protein, which showed 100% identity with YP_498675.1 in the HHPred server. The 3D model was viewed by USCF Chimera 1.16 and shown in (Fig. 5).

**Fig. 6:**
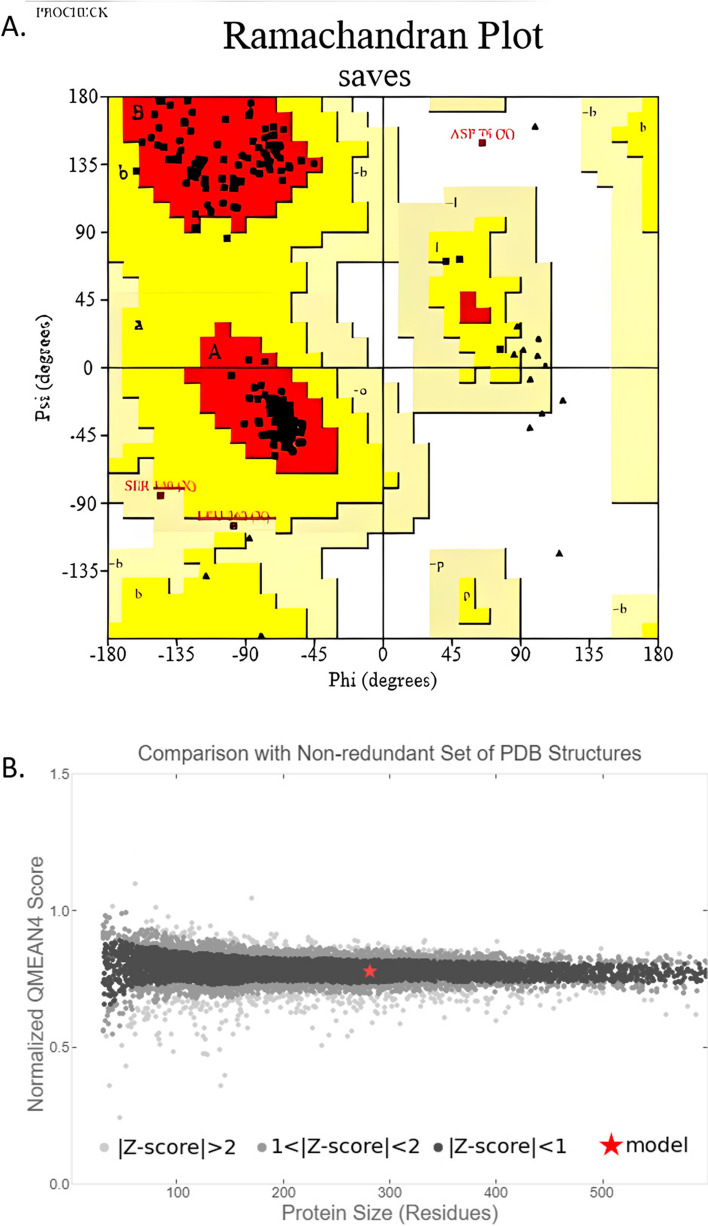
Model quality assessment. **A** Ramachandran plot of the model structure validated by PROCHECK server. Here, 96.7% amino acid residues covered the most favored regions (A, B, L). **B** Graphical representation of QMEAN result of the model structure. Here, *Z* score of the anticipated model was 0.14 (indicates good agreement between the model structure and experimental structure of similar size)

PROCHECK, Verify 3D, QMEAN, and ERRAT were used to evaluate the quality of our modeled 3D structure. To validate protein models, the program PROCHECK employs several evaluation metrics, including the generation of a Ramachandran plot and the analysis of torsion angles, surface areas, bond angles, and atomic distances. These calculations play a crucial role in assessing the structural integrity and accuracy of protein models [40]. According to PROCHECK result, the most favored region in the “Ramachandran plot” had 96.7% of amino acid residues, with 2%, and 0.8% residues in additional allowed and generously allowed regions, respectively, indicating that the model was reliable and of good quality (Table 3 and Fig. 6A). ERRAT was used to assess the model’s reliability by analyzing the statistics of non-bonded interactions between distinct atom types based on characteristic atomic interactions. The template’s overall quality factor was found to be 87.546, indicating a structure with good high resolution. According to the Verify 3D tool, 100% of residues had an averaged 3D (atomic model)–1D (amino acid) score ≥ 0.2, indicating that these structures were compatible and excellent. The model was placed into the dark gray zone by the QMEAN tool, with a QMEAN4 value of 0.14. This score is considered good since the threshold value for the QMEAN score, which ranges from 0 to 1, falls within the acceptable range (Fig. 6B).

**Table 3:** Ramachandran plot statistics of the hypothetical protein (YP_498675.1)

Statistics	Number of AA residues	Percentage (%)
Residues in the most favored regions (A, B, L)	236	96.7%
Residues in the additional allowed regions (a, b, l, p)	5	2%
Residues in the generously allowed regions (~ a, ~ b, ~ l, ~ p)	2	0.8%
Residues in disallowed regions	1	0.4%
Number of non-glycine and non-proline residues	244	100%
Number of end-residues (excl. Gly and Pro)	2	
Number of glycine residues (shown as triangles)	24	
Number of proline residues	11	
Total number of residues	281

#### Active site determination

**Fig. 7:**
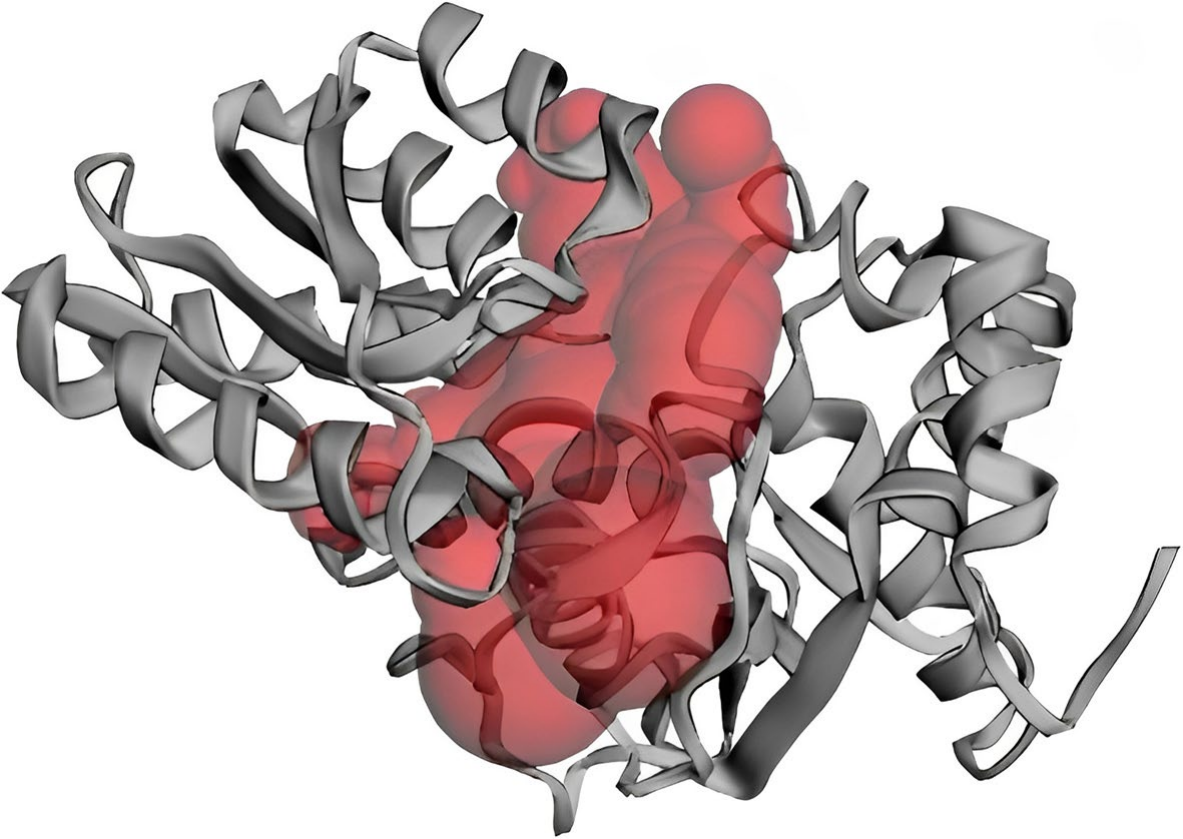
Determination of active site of YP_498675.1 using the CASTp server. The largest active site was found in the areas with 1198.087 and volume of 1046.218 amino acids

The identification and characterization of active site residues are key steps in the design of a drug or inhibitor. The CASTp server was used to assess the active site of the model structure, as well as to determine the active site amino acid residues. The top active sites of the model protein were determined in one of the largest pockets using the area of 1198.087 and the volume of 1046.218 amino acids. According to CASTp prediction, the model protein’s active residues are shown in (Table 4 and Fig. 7).

**Table 4:** CASTp analysis result: Active site of amino acid residues. Here, A.A, amino acid; SeqID, position of AA in protein sequence

A.A	SeqID	A.A	SeqID	A.A	SeqID	A.A	SeqID	A.A	SeqID
MET	1	THR	59	TYR	107	PRO	176	PRO	253
LYS	2	ASN	60	ASN	112	ILE	177	ASP	254
PRO	5	ASP	78	HIS	116	ASN	178	ARG	255
GLU	27	HIS	80	PRO	138	ARG	179	GLY	256
SER	28	GLY	82	VAL	139	GLU	180	ASP	257
THR	29	TYR	83	SER	140	LEU	181	ARG	258
SER	30	LEU	84	THR	141	PRO	182	TYR	259
GLY	31	MET	85	THR	142	GLY	183	LEU	260
ASN	32	ARG	87	GLY	143	ILE	184	ASP	261
LEU	33	ILE	88	SER	144	GLY	185	LEU	262
ASP	53	VAL	91	ILE	145	ALA	186		
LYS	55	GLN	92	LYS	167	SER	187		
ILE	56	TRP	103	GLY	168	ARG	188		
SER	57	ASN	105	SER	169	SER	227		
PRO	58	GLN	106	VAL	170	ILE	251

#### Energy minimization result

The energy of the predicted protein’s three-dimensional structure was minimized by YASARA force field minimizer. The energy was reduced to − 85,376.8 kj/mol from − 150,380.7 kj/mol after energy minimization. After energy minimization, the final score turned from − 1.46 to − 0.21, suggesting a more stable structure.

## Discussion

*S. aureus* is a gram-positive, facultative aerobe, tiny, spherical, or non-motile cocci that do not produce spores and are catalase and coagulase positive. *S. aureus* is a significant human and animal pathogen because it produces exotoxins called superantigens (SAgs). The spectrum of SAg-mediated diseases included from relatively benign food poisoning to life-threatening toxic shock syndrome (TSS). The major secreted SAgs of *S. aureus* include TSS toxin 1 (TSST-1) and enterotoxin (SE) serotypes A to Q, excluding F [50, 51]. This is one of the most widespread bacterial pathogens, responsible for hundreds of thousands to millions of more serious, invasive infections each year, and an uncountable number of simple skin infections [52, 53]. It is a leading causative agent in pneumonia and other respiratory tract infections, surgical site, prosthetic joint, and cardiovascular infections, as well as nosocomial bacteremia [54]. Over the last two decades, scientists have worked to develop a vaccine against *S. aureus*, but in clinical trials, no vaccine candidates have been found to be effective. Characterization of HPs YP_498675.1 of *S. aureus* can aid in understanding bacterial metabolic regulations, formulating disease control strategies, and developing effective therapeutics. Various computational resources were employed in this study to characterize the hypothetical protein YP_498675.1 of *S. aureus* from structural and functional aspects. The physiochemical properties’ analysis revealed that the protein consists of 281 amino acid sequence, have a molecular weight of 30,872.44, the grand average of hydropathicity (GRAVY) score of − 0.119, and a theoretical PI of 5.78 (Table 1). The Protparam tool calculates the extinction coefficient of HP at 280 nm, ranging from 33,015 to 32,890 M^−1^ cm^−1^. This coefficient is valuable for quantitative analysis of protein interactions, including interactions with ligands and other proteins [49]. In our study, we employed CELLO, a subcellular location prediction tool, to analyze the hypothetical protein YP_498675.1. The results from CELLO indicated that the protein predominantly localizes to the cytoplasm, which aligns perfectly with the findings of PSoRTb, SOSUI, and PSLpred. Furthermore, this observation was reinforced by the ProtParam GRAVY index (− 0.119), which suggested that the protein possesses a hydrophilic nature. Given that hydrophilic proteins are commonly found in the cytoplasmic compartment within cellular environments, the CELLO prediction further substantiates our findings. The analysis of the protein’s secondary structure reveals the prevalence of extended strand, beta turn, alpha helix, and random coil. The analyzed hypothetical protein (YP_498675.1) of this study is predicted to have the pyridoxal phosphate (PLP)-dependent enzyme motif. PLP-dependent enzymes in *S. aureus* may play crucial role in the development of skin diseases. PLP is the active form of vitamin B6 and serves as a cofactor for numerous enzymes involved in different metabolic pathways. One of the crucial involvement of PLP-dependent enzymes in cell wall synthesis that can influence the integrity and structure of the bacterial cell envelop, which is vital for the *S. aureus* colonization and evasion of host immune responses. Furthermore, it has been found that some PLP-dependent enzymes can produce metabolites or by-products that directly influence host immune response and contribute to tissue damage and progression of skin diseases [55, 56]. Further research is necessary to fully comprehend the specific role of the hypothetical protein YP_498675.1 and its association in the context of *S. aureus* skin infections.

The virulence and survival of pathogenic bacteria such as *S. aureus* is depended on PLP. The conserved protein domain of the hypothetical protein YP_498675.1 is found to be 2, 3-diaminopropionate biosynthesis protein SbnA, a protein of the staphyloferrin B biosynthesis operon. It is known that SbnA is a PLP-dependent enzyme and actively involved in many cellular processes and biosynthesis of natural products. SbnA and SbnB are encoded by the staphyloferrin B biosynthetic gene cluster and are implicated in L-2, 3-diaminopropionic acid (L-Dap) biosynthesis. SbnA and SbnB together appear to synthesize 2, 3-diaminopropionate, a precursor of certain siderophores and other secondary metabolites [57]. Further analysis by protein BLAST 2103 (BLASTp) against the non-redundant database revealed that YP_498675.1 has up to 100% sequence similarity with other 2,3-diaminopropionate biosynthesis protein SbnA of *S. aureus* and other related organisms (Table 2). The results of protein domain and BLASTp analysis clearly indicate that hypothetical protein YP_498675.1 may have an important functional role in cellular metabolism of *S. aureus*.

Understanding the three-dimensional structure of proteins is crucial for comprehending their interactions, functions, and localization. The most widely employed method for predicting protein structures is homology modeling. In our current research, we utilized homology modeling to propose the initial 3D structure of a hypothetical protein in *S. aureus* called YP_498675.1. This predicted structure will offer valuable insights into the protein’s structure and function, enabling further exploration of drug design and protein interactions [58]. The tertiary structure of the YP_498675.1 was developed from HHpred server and the quality of the model was assessed by evaluation software like Verify 3D, PROCHECK, ERRAT, and QMEAN. It has been estimated that about 96.7% amino acid residues of the model 3D structure covered the most favored region in Ramachandran plot, which depicts the model quality as valid (Fig. 6A). The result of QMEAN4 server (Fig. 6B) showed that the *Z* score of the anticipated model was 0.14, which also denotes a good quality model. After YASARA energy minimization process, the 3D structure of hypothetical protein YP_498675.1 became more stable. Prediction of active-sites residues by CASTP server is a very important step in the design of a drug or inhibitor. These active site residues can be identified and characterized to learn more about the protein’s enzymatic activity, binding properties, and probable involvement in numerous biological processes. CASTp is a database server that is capable of identifying and characterizing distinct regions on proteins. It can determine the boundaries of these regions, calculate their sizes, and analyze their dimensions [49]. These regions encompass pockets on the protein’s surface as well as internal voids within the protein structure. By utilizing CASTp, the primary active sites of the protein model were precisely identified, with sizes varying between 1198.087 in terms of area and 1046.218 in terms of volume. In CASTp analysis, one largest pocket was found as active sites with solvent-accessible (SA) surface area of 1198.087 and volume of 1046.218 amino acids. Overall, the CASTp analysis advances our understanding of the structure–function link of the protein and opens the door for further research into the precise molecular pathways involved. 

## Conclusion

Our study delved into the in silico structural and functional annotation of a novel hypothetical protein YP_498675.1, derived from *S. aureus*. Through a meticulous computational analysis, we gained valuable insights into the characteristics and functionalities of this protein. Functional annotation unraveled potential functional domain (Try-synth-beta_ II) and motif (PLP-dependent enzyme), providing clues about its putative biological roles. By thoroughly examining YP_498675.1, we have expanded our knowledge regarding its putative involvement in vital cellular processes and interactions. Our research advances knowledge of the genetic and proteomic profile of *S. aureus*, identifying putative targets for development of a drug or vaccine against this pathogenic bacterium. While this study represents a crucial initial steps towards the functional significance of YP_4986675.1, it is warranted to conduct further experimental validation and functional characterization to confirm the predicted structural and functional attributes. Nonetheless, our comprehensive in silico analysis lays a solid foundation for future research, offering valuable insights into the potential roles of YP_4986675.1 and its implications with the realm of *S. aureus* physiology and pathogenesis.

## Data Availability

All data analyzed during this study are included in this article.
